# Evaluating handgrip strength and functional tests as indicators of gait speed in older females

**DOI:** 10.3389/fspor.2025.1497546

**Published:** 2025-01-27

**Authors:** Valentina Muollo, Samuel D’Emanuele, Laura Ghiotto, Doriana Rudi, Federico Schena, Cantor Tarperi

**Affiliations:** Department of Neuroscience, Biomedicine and Movement Sciences, University of Verona, Verona, Italy

**Keywords:** older women, physical function, mobility limitation, gait speed, grip strength, cross sectional study

## Abstract

**Introduction:**

With aging, females often experience greater declines in functional capacity [e.g., gait speed (GS)] compared to males, highlighting the need for sex-difference considered in screening and intervention planning. In certain contexts, assessing GS may not be feasible. Handgrip strength (HGS) commonly used as a surrogate measure for physical performance, also serves as an indirect indicator of muscle strength in the lower limbs. This cross-sectional study aims to investigate the associations between HGS and common functional tests and to determine the optimal cut-off values for these tests in assessing GS.

**Methods:**

142 community-dwelling older females aged 60–80 years old (mean age: 75 ± 6 years) were evaluated with HGS, the 30-second arm curl (30 s-AC), 30-second chair stand (30 s-CS), the Short Physical Performance Battery (SPPB), and the 8-foot Up & Go (8-UG) test. Pearson's correlation (r) was used to assess the strength of associations between HGS and functional variables, while multiple linear regression models identified determinants of GS. Receiver operating characteristic (ROC) curves were employed to evaluate the effectiveness of various tests in detecting slow GS (<1.0 m/s), by means of the area under the curve (AUC), sensitivity, and specificity.

**Results:**

HGS showed positive significant (*p* < 0.001) associations with 30 s-AC (r = 0.499), SPPB (r = 0.447), and 30 s-CS (r = 0.329). Standardised coefficients of the linear models were: 30 s-AC (*β*=0.593), 30 s-CS (*β*=0.513), 5-CS (*β*=−0.431), and HGS (*β*=0.475) (all *p* < 0.001). ROC analysis revealed the following results: 30 s-AC (AUC = 0.80, cut-off=∼16 repetitions, sensitivity 83%, specificity 36%), 30 s-CS (AUC = 0.74; cut-off=∼13 repetitions, sensitivity 78%, specificity 64%), and 5-CS (AUC = 0.75, cut-off = 10.0 s, sensitivity 81%, specificity 57%), HGS (AUC = 0.73, cut-off=∼20 kg, sensitivity 79%, specificity 46%).

**Discussion:**

We found that HGS was moderately-to-weakly associated with functional outcomes in older females, indicating that it may not reflect the overall body functional capacity. Despite similar AUCs across all tests, the 30 s-CS and 5-CS showed a better balance of sensitivity and specificity, making them potential indicators of slow GS compared to HGS and 30 s-AC.

## 1 Introduction

The aging process involves complex physiological, metabolic, structural, and functional changes that result in a progressive reduction of mobility, independence, and overall quality of life (1, 2). A significant contributor to this decline is the loss of muscle mass, which diminishes muscle strength and power, impairing the ability to perform daily activities such as walking and climbing stairs (3, 4). These changes are associated with an increased risk of falls, frailty, and reduced life expectancy, particularly in older adults who experience more pronounced functional impairments.

Sex differences play a crucial role in how these age-related declines manifest. In females, these declines are exacerbated by a significant reduction in sex hormone concentrations following menopause, coupled with an increased risk of osteoarthritis and reduced baseline muscle strength (5). Consequently, females experience a slightly greater decline in performance compared to males, particularly after the age of 60. Among the measurable indicators of functional decline, gait speed is particularly valuable. Gait speed is strongly associated not only with life expectancy and adverse health outcomes but also with other measures such as handgrip strength (HGS), lower limb strength, and balance assessments (6–8). Research indicates that females walk slower than males by approximately 0.054 m/s on average. While this difference may seem small, it has practical implications, as slower gait speed is associated with reduced functional capacity, increased risk of falls, and greater dependence on daily activities, especially among older adults (9). This highlights the need to account for sex-specific differences when assessing mobility and planning interventions. However, direct assessments of gait speed may not always be feasible, especially in contexts with space or cognitive constraints (10).

In such cases, alternative, easy-to-administer measures that approximate gait speed are needed. HGS has emerged as a practical proxy for muscle strength and functional abilities, given its simplicity and accessibility (6, 7, 11). HGS is commonly used to estimate lower limb strength and overall physical performance, including mobility. However, while muscle weakness is a primary contributor to slow gait speed in older populations (12, 13), other factors such as balance impairments, neurological conditions, and reduced coordination also influence mobility (14). The relationship between HGS and gait speed is not fully understood, particularly when considering sex-related differences. This raises the need to further evaluate whether HGS can effectively classify individuals with slow gait speed, especially in female populations.

Functional tests such as the chair stand [in five-repetition (5-CS) or in thirty-second (30 s-CS)] are also employed to evaluate functional status in older adults (7). Compared to HGS, the chair stand test can assess a broader range of factors, including sensorimotor integration, psychological readiness, and balance (15). However, often the chair stand might be perceived as more challenging than the HGS, with the consequence that older individuals might not be able to complete the test appropriately (15). This occurrence appears to be particularly relevant in the female population. In fact, females generally take longer than males to complete the chair stand test, and this is likely due to inferior lower extremity muscle strength and a higher prevalence of osteoarthritis (16).

In light of the aforementioned considerations, it becomes crucial to establish specific cut-off values for different functional tests to classify individuals with low gait speed. Although several studies have focused on determining accuracy and cut-offs for HGS (10, 17), and a few have examined the 5-CS (8, 16), there is a lack of comprehensive research comparing multiple functional assessments [i.e., HGS, 5-CS, 30 s-CS and thirty-second arm curl (30 s-AC)] to discriminate their effectiveness in predicting low gait speed in specific populations.

This study aims to fill this gap by focusing on community-dwelling older females. Given the widespread use of HGS in clinical settings, the first goal of the study was to investigate the associations between HGS and other measures of upper and lower limbs strength (e.g., 30 s-AC, 5-CS, 30 s-CS) and overall physical performance (Short Physical Performance Battery ((SPPB), which includes the gait speed test) and 8-foot Up & Go (8-UG)). We hypothesized that HGS would demonstrate weaker associations with lower body strength (e.g., 5-CS, 30 s-CS) and performance (gait speed).

A second goal of the study was to establish optimal cut-off values for the most common functional tests (i.e., HGS, 5-CS, 30 s-CS and 30 s-AC) in the classification of individuals with low vs. adequate gait speed. We hypothesized that functional tests incorporating lower extremity strength (e.g., 5-CS and 30 s-CS) would offer better sensitivity and specificity in identifying individuals with slow gait speed compared to HGS or 30 s-AC, reflecting the greater relevance of these measures to walking ability.

## 2 Materials and methods

### 2.1 Study design and participants

This cross-sectional study was carried out in the Northwest of Italy, and all procedures were conducted in accordance with the Declaration of Helsinki. The study protocol received approval from the Ethical Committee of the University of Verona (registration number #04/2024).

Participants were recruited through a combination of community outreach efforts, including flyers posted at local senior centres, University, health clinics, and community events, as well as word-of-mouth referrals. Eligible participants were community-dwelling older females aged 60–80 years who lived independently and were confirmed to be free from severe physical and cognitive impairments or arthritis through self-report and screening assessments conducted during recruitment. To ensure the reliability of the results, females who were unable to adhere to the testing sessions due to cognitive or physical limitations were excluded. Participants were interviewed before the testing sessions to confirm that they were feeling well and had not taken any substances, such as pain medications or caffeine, that might affect their performance. Those who self-reported feeling unwell or reported the intake of such substances were excluded. Prior to participation, all participants signed written informed.

### 2.2 Anthropometric indices

Body mass and stature were assessed respectively with an electronic scale and a Harpenden Stadiometer to calculate the BMI [as weight (kg)/height (m)^2^] of each participant (18). All the tests were performed in a single session.

### 2.3 Functional evaluations

The maximal strength of the upper limbs was evaluated with a handgrip dynamometer (Lafayette Instrument, Lafayette, IN, USA). Participants were instructed to stand upright with their arm, forearm, and wrist in a neutral position and apply maximum hand grip pressure for a maximum of five seconds. Three trials using the dominant arm were conducted, each separated by a one-minute rest period (19). The highest peak expressed in absolute units was used for the analysis (19).

The 30 s-AC and 30 s-CS tests were collected to measure participants' muscular endurance strength (20). In the 30 s-AC test, participants performed as many flexion-extension movements of their dominant elbow with a 2.3 kg dumbbell within a 30-s time frame. In the 30 s-CS test, participants performed the maximum number of full stand-ups from a seated position within a 30-s time frame, ensuring proper form with arms crossed on their chest and achieving a full range of motion (i.e., standing fully upright). Any repetitions that did not meet these criteria were not counted. Global physical performance was evaluated using the SPPB test, which consists of a 4-m walk at customary gait speed, a 5-CS, and three standing balance tests (side-by-side, semi-tandem, and tandem) (21). Both the gait speed and 5-CS tests were performed twice, with the best trial recorded, while the three standing balance tests were performed only once, following the standardized guidelines. During each test, the experimenter used a stopwatch to record the time, assigning a score from zero to four points based on recommended cut-off values. The maximum score achievable on the SPPB 12 points, calculated by summing the scores from each category (21). Gait speed, a core outcome of this study, was assessed as part of the SPPB.

Finally, the 8-UG test was administered to assess participants' mobility and functional capacity. The test began with participants seated in the chair (∼46 cm seat height), with their back against the chair and arms resting on the armrests (20). Upon hearing the “*go*” signal, participants were instructed to stand up, walk at a comfortable and safe pace to a marked line on the floor 2.8 meters away, turn around, return to the chair, and sit down again. No physical assistance was provided during the test. In the end, the experimenter used a stopwatch to collect the time in seconds to complete the task. Participants completed two trials, and their best performance from the trials was utilized for the analysis.

### 2.4 Statistical analysis

The data were summarized as mean ± standard deviation. Normality was assessed using the Shapiro-Wilk test. To evaluate the association between HGS and functional variables, Pearson's correlation coefficient (r) was used based on the normality assumption. The strength of the associations was categorized as follows: <0.4 indicating a *weak* association, 0.4–0.6 representing a *moderate* association, and >0.6 indicating a *strong* association (22). Additionally, a simple linear regression analysis was conducted to explore which outcome more accurately predicted gait speed (i.e., the dependent variable in each of the models). Seven linear models were created, and the coefficients of the linear relationship were retrieved. The slope of the relationship, when data was normalised by the standard error, returned the *β* coefficient for each model. For the different models, the following independent variables were considered: model (1) Age, model (2) BMI, model (3) 30 s-AC, (4) 30 s-CS, (5) Tandem, (6) 5-CS, (7) HGS. A *p*-value for each linear regression model was reported. Finally, receiver operating characteristic (ROC) curves were generated to evaluate the ability of HGS, 5-CS, 30 s-CS and 30 s-AC tests to discriminate between participants with slow gait speed (<1.0 m/s, positive outcome) and those without (>1.0 m/s, negative outcome) (23). Area under the curve (AUC) values were calculated and interpreted as follows: ≤0.6 (*fail*), 0.6–0.7 (*poor*), 0.7–0.8 (*fair*), 0.8–0.9 (*good*), 0.9–1.0 (*excellent*) (24). Optimal cut-off values were determined based on the point that provided the best balance between sensitivity (i.e., the true positive rate) and specificity (i.e., the true negative rate) for identifying participants with low GS.

Analysis was performed using SPSS statistics software (Version 23.0, Chicago, IL, USA), and the statistical significance was set at *p* < 0.05.

### 2.5 Statistical power calculation

A statistical power calculation was conducted using G*Power 3.1 software. In a study (25) conducted on a cohort of 209 older individuals (mean age 75 years), a positive association between HGS and gait speed was reported in the group of females (*r* = 0.30). Drawing from these findings, we conducted a sample size analysis for Pearson's correlation coefficient in our study, aiming for a statistical power of 0.8 and a significance of 0.05. The analysis revealed that a minimum of 84 participants would be required to detect a significant association between HGS and gait speed. This sample size estimation accounts for a weak effect size within the population.

## 3 Results

The main characteristics of the study participants are presented in Table 1. A total of 142 older females were included in the study, with 16 participants (11%) classified as having obesity (8 with class I and 3 with class II obesity). Additionally, 71 participants (approximately 50%) were classified as having low gait speed (i.e., GS < 1 m/s).

**Table 1:** Characteristics of study participants.

Variables	*n* = 142
Age (years)	75.0 ± 6.3
Height (m)	1.59 ± 0.06
Weight (kg)	63.8 ± 10.6
BMI (kg/m^2^)	25.4 ± 4.0
30 s-AC (n)	15.8 ± 3.7
30 s-CS (n)	13.1 ± 3.7
SPPB (score)	10.7 ± 1.6
Semi-tandem (s)	9.9 ± 0.8
Tandem (s)	9.2 ± 2.4
5-CS (s)	11.9 ± 3.6
GS (m/s)	1.01 ± 0.23
8-UG (s)	7.2 ± 2.2
HGS (kg)	21.4 ± 5.5

Data are presented as mean ± SD.

Abbreviations: BMI, body mass index; 30 s-AC, 30-second arm curl; 30 s-CS, 30-second chair stand; SPPB, short physical performance battery; 5-CS, chair stand in five-repetition; GS, gait speed; 8-UG, foot up and go; HGS, handgrip strength.

### 3.1 Association analysis between handgrip strength and functional outcomes

The results of the association analyses between HGS and other variables are presented in Table 2. A moderate association was found between HGS and 30 s-AC and SPPB total score while showing a weak association with tandem and 30 s-CS (all *p* < 0.001). No significant associations were observed between HGS and age, BMI, 5-CS, gait speed, and 8-UG (*p* > 0.05).

**Table 2:** Coefficient correlation between handgrip strength and other variables.

Variables	r		*p*-value
Age (years)	−0.423	weak	0.343
BMI (kg/m^2^)	−0.084	weak	0.070
30 s-AC (n)	0.499	moderate	<0.001
30 s-CS (n)	0.329	weak	<0.001
SPPB (score)	0.447	moderate	<0.001
Tandem (s)	0.238	weak	<0.001
5-CS (s)	−0.396	weak	0.082
GS (m/s)	0.475	moderate	0.320
8-UG (s)	−0.380	weak	0.500

Abbreviations: BMI, body mass index; 30 s-AC, 30-second arm curl; 30 s-CS, 30-second chair stand; SPPB, short physical performance battery; 5-CS, chair stand in five-repetition; GS, gait speed; 8-UG, foot up and go.

### 3.2 Linear regression analysis between gait speed and functional outcomes

The results of the linear regression analysis are summarized in Table 3. In model 3, examining the relationship between 30 s-AC and gait speed, the standardized *β* coefficient was 0.593. This suggests that for each standard deviation increase in 30 s-AC (approximately four repetitions), there was a corresponding increase of 0.14 m/s in gait speed. Similarly, in model 4 for the relationship between 30 s-CS and gait speed, the standardized *β* for the 30 s-CS was 0.513, indicating that each one standard deviation increase in the 30 s-CS (approximately four repetitions) was associated with a 0.12 m/s increase in gait speed. In model 5, focusing on the relationship between tandem and gait speed, the standardized *β* for tandem was 0.338, meaning that each standard deviation increase in tandem (approximately 2.4 s) corresponded to a 0.08 m/s increase in gait speed.

**Table 3:** Linear regression models between gait speed and different functional tests.

Models	Variables	Standardized coefficient		*p*-value
		*β*	t	
Model 1 (R^2^ = 0.129)	Constant	−0.359	9.296	<0.001
	Age		−4.548	0.093
Model 2 (R^2^ = 0.023)	Constant	−0.150	9.411	<0.001
	BMI		−1.691	0.093
Model 3 (R^2^ = 0.352)	Constant	0.593	6.309	<0.001
	30 s-AC		8.711	<0.001
Model 4 (R^2^ = 0.263)	Constant	0.513	9.744	<0.001
	30 s-CS		7.064	<0.001
Model 5 (R^2^ = 0.114)	Constant	0.338	9.917	<0.001
	Tandem		4.248	<0.001
Model 6 (R^2^ = 0.186)	Constant	−0.431	9.411	<0.001
	5-CS		−1.691	<0.001
Model 7 (R^2^ = 0.226)	Constant	0.475	22.198	<0.001
	HGS		−5.651	<0.001

Abbreviations: BMI, body mass index; 30 s-AC, 30-second arm curl; 30 s-CS, 30-second chair stand; 5-CS, chair stand in five-repetition; HGS, handgrip strength.

Conversely, in model 6 for the relationship between the 5-CS and gait speed, the standardized *β* for 5-CS was −0.431, indicating that each standard deviation increase in 5-CS (approximately 3.6 s) led to a decrease of 0.1 m/s in gait speed. In model 7, examining the relationship between HGS and gait speed, the standardized *β* for HGS was 0.475, indicating that each standard deviation increase in HGS (approximately 5.5 kg) corresponded to a 0.1 m/s increase in gait speed. No significant results were observed in models 1 and 2.

### 3.3 Determining optimal cut-offs for functional tests: ROC curve analysis

**Figure 1:**
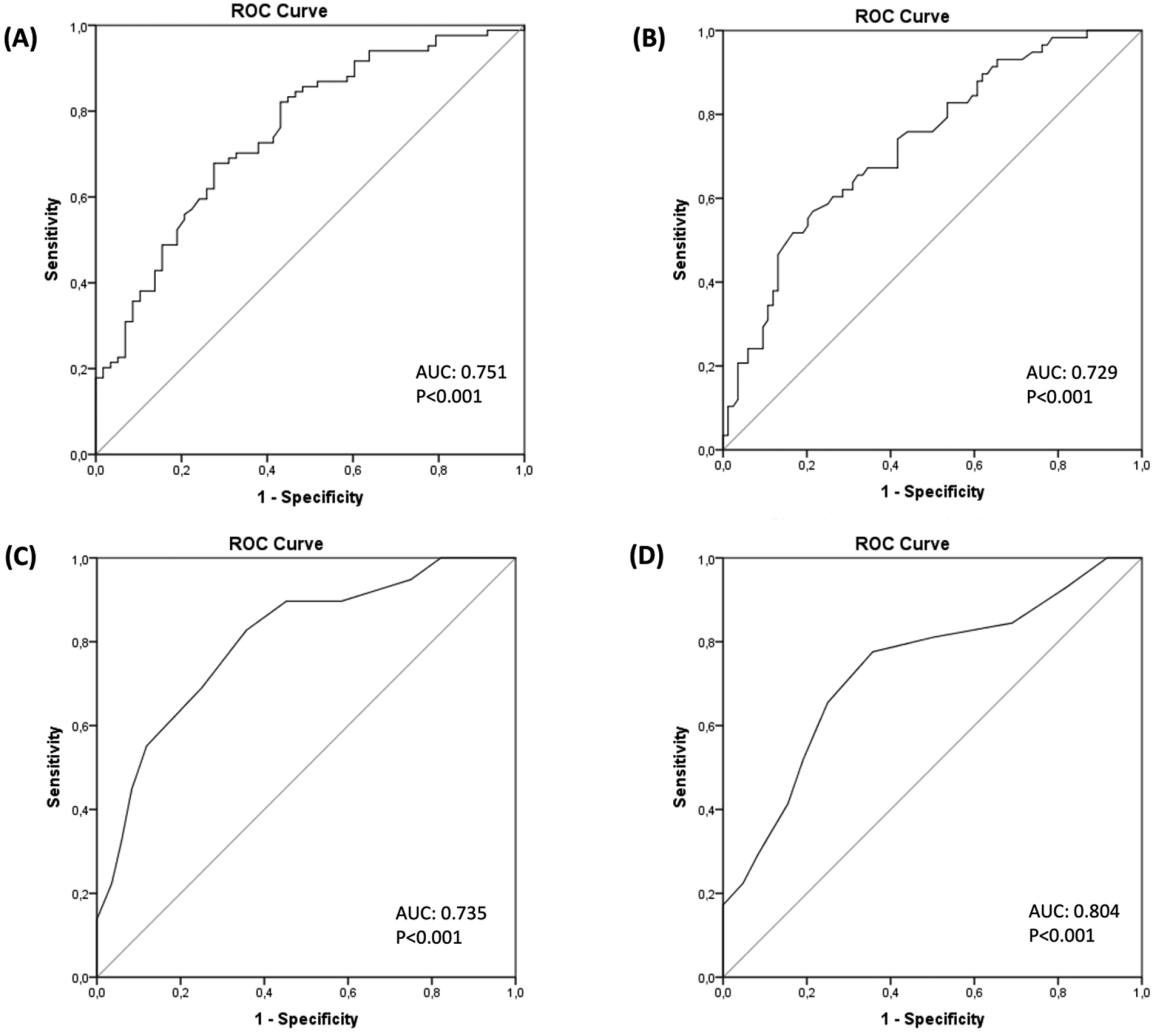
Cut-offs identification via ROC curve analysis for functional tests. Receiver operating characteristics curve plots compare how the 5-Chair Stand test [panel **(A)**], Handgrip Strength [Panel **(B)**], 30-second Chair Stand [Panel **(C)**], 30-second Arm Curl [Panel **(D)**] tests allow the identification of older adults with low gait speed. ROC, receiver operating characteristic.

Figure 1 displays the predicted capacity of different functional tests for low gait speed. For the detection of slow gait speed, a 5-CS cut-off value of 10.4 s exhibited a sensitivity of 81% and a specificity of 57%, with an AUC of 0.75 (classified as fair test) [Panel (A)]. This means that using a cut-off of 10.4 s for the 5-CS, we can expect 81% of positive outcomes (indicating slow gait speed) to be correctly classified, while 43% of negative outcomes would be incorrectly identified as positive. Similarly, a HGS cut-off value of 19.95 kg showed a sensitivity of 79% and a specificity of 46%, with an AUC of 0.73 (also classified as a fair test) [Panel (B)]. This implies that using a cut-off of ∼20 kg for the HGS, we anticipate correctly classifying 79% of positive outcomes (indicating slow gait speed). In contrast, 54% of negative outcomes would be incorrectly identified as positive.

In the case of the 30 s-CS, a cut-off value of 12.5 repetitions had a sensitivity of 78% and a specificity of 64%, yielding an AUC of 0.74 (classified as a fair test) [Panel (C)]. This suggests that with a cut-off of 12.5 repetitions for the 30 s-CS, we anticipate that 78% of positive outcomes (indicating slow gait speed) would be correctly classified as positive, while 36% of negative outcomes would be incorrectly identified as positive. Finally, for a 30 s-AC test with a cut-off value of 15.5 repetitions, a sensitivity of 83% and a specificity of 64% were observed, resulting in an AUC of 0.80 (classified as a good test) [Panel (D)]. This indicates that by using a cut-off of 15.5 repetitions for the 30-AC, 83% of positive outcomes (indicating slow gait speed) would be correctly classified as positive, while 36% of negative outcomes would be incorrectly identified as positive.

## 4 Discussion

This study focused on community-dwelling older females and our primary objective was to investigate the associations between HGS and gait speed, as well as other functional fitness outcomes. For the first time, we conducted a comprehensive comparison of multiple functional tests (HGS, chair stand, and arm curl tests) to assess their relative contributions to functional capacity and gait speed, focusing on a large sample of older females. Additionally, we aimed to identify key functional factors influencing gait speed and establish optimal cut-off values for these tests, especially for cases where individuals are unable to walk or when the space available to perform the test is limited.

The correlation analyses returned significant associations between HGS and various functional outcomes. Notably, HGS showed moderate associations with 30 s-AC and SPPB total score. However, weak associations were observed with tandem and 30 s-CS, while no significant associations were found with the 8-UG and 5-CS. As expected, these findings suggest that HGS may not always fully capture lower body function. While HGS is a useful indicator of overall muscle strength, it primarily reflects upper body strength and may not directly capture the more specific lower body functional impairments that influence gait performance. Key functional factors directly affecting gait performance include lower limb strength, balance, and coordination (14). Among the measures used in this study, tests like the 5-CS and 30 s-CS tests are more directly linked to gait performance, as they assess lower extremity strength, dynamic balance, and coordination, all of which are critical components for effective walking (26). These tests are more directly related to the muscles and systems responsible for gait, whereas HGS, though important, may not provide a complete picture of the functional limitations impacting walking speed.

A recent review (27) presented conflicting results concerning the relationship between HGS and overall physical performance, particularly in terms of lower muscle strength. The limited number of studies explicitly focused on older females, coupled with the different tests used, makes direct comparisons challenging. For instance, Wisnowska et al. (25) identified weak associations between HGS with age (*r* = −0.18, *p* = 0.053), chair stand performance in seconds (*r* = −0.27), and 10-m gait speed (*r* = 0.30), with no significant associations observed with BMI. While the findings for age and HGS did not reach statistical significance (trend with *p* = 0.053), they suggest a potential relationship that warrants further exploration. Similarly, in our study, we observed weak but nonsignificant associations between HGS and age and BMI, alongside a significant association with 30 s-CS performance. Together, these findings underscore the variability in how HGS relates to different functional outcomes, which may be influenced by population characteristics or test-specific factors. Similarly, another study (28) involving older adults of both sexes, found weak or no associations between HGS and the timed up-and-go test under different conditions (acoustic evaluation, pre-recorded sound signal, and visual assessment). Our findings corroborate some of these results, underscoring the importance of exercising caution when using HGS alone as a surrogate for more complicated measures of arm and leg strength (7) or performance (27). Tasks like rising from a chair involve muscles beyond those involved in gripping, contributing to the observed weak association between HGS and lower limb performance (29). Consequently, the absence of robust associations between HGS and lower limb performance should not be surprising (29), and further supports the need to perform multiple tests to achieve more accurate assessments in older females.

Simple linear regressions were chosen to clearly illustrate the direct impact of each functional test on gait speed. This approach allowed us to isolate the influence of each variable without the complications of multicollinearity that may arise in multiple regression models. Although future studies could use multiple regression to explore these relationships in greater depth, we opted for simpler models to avoid increasing the R^2^ value unnecessarily by including variables of unknown importance. The analyses revealed a significant relationship between functional outcomes and gait speed, with increases in both 30 s-AC and 30 s-CS tests being strongly associated with corresponding improvements in gait speed. Specifically, we observed that an increase of approximately four repetitions in the 30 s-AC and 30 s-CS tests was associated with a gait speed increase of 0.14 m/s and 0.12 m/s, respectively. Similarly, improvements in HGS by around 5.5 kg and a reduction in the 5-CS test time by 3.6 s were associated with a gait speed increase of 0.1 m/s each. To put these results in perspective, a change in gait speed between 0.05–0.1 m/s is considered clinically meaningful (6), while the clinically meaningful change for the 5-CS and HGS are 2.3 s (6) and 5.0–6.5 kg, respectively (30). Our study contributes to the growing body of literature by confirming these associations and providing specific quantitative relationships that can inform clinical assessments and interventions.

The prevalence of slow gait speed is highly dependent on the cut-off values adopted, such as 0.8 m/s vs. 1.0 m/s (31). Setting the cut-off value to 0.8 m/s may lead to an underestimation of individuals with low gait speed who are at increased risk of adverse health outcomes, including enduring lower extremity limitations, hospital admissions, and mortality (32, 33). In this study, we used ROC curve analysis to compare the predictive capacity of different functional tests in detecting slow gait speed. In ROC analysis, a key consideration is whether to prioritize sensitivity or specificity. Sensitivity is prioritized when the goal is to correctly identify individuals at risk (minimizing false negatives), whereas specificity is favoured when avoiding false positives is critical. Given the limited literature on sensitivity and specificity cut-offs for HGS and other functional tests in older populations, calculating an exact sample size for ROC analysis was challenging. Future studies should consider these trade-offs in their design to maximize the clinical relevance of the functional measures being evaluated. Previous studies (10, 17) investigating the link between HGS and gait speed in older females reveal variations in cut-off values for impaired walking speed. Delinocente et al. (17) established a cut-off value of ∼21 kg for HGS with 58.6% sensitivity and 72.9% specificity, while Felix et al. (10) found a similar cut-off value with higher sensitivity (100%) and specificity (91%). However, in our cohort, we observed a slightly lower cut-off value of ∼20 kg for HGS, resulting in 79% sensitivity, 54% specificity, and an AUC of 0.73. These discrepancies in the cut-off values between studies indicate potential variability in the HGS-walking speed association among older females, influenced by factors like age and sample size. Another study (16) investigated the relationship between walking speed and the 5-CS test in older females. Using a walking speed cut-off value of 0.1 m/s, they determined a 5-CS cut-off of 11.04 s with 68% sensitivity, 67% specificity, and an AUC of 0.74. In our cohort, we identified a similar 5-CS cut-off of 10.4 s, with 81% sensitivity, 57% specificity, and an AUC of 0.75. These findings suggest the potential utility of the 5-CS test in assessing functional performance, particularly regarding gait speed in older females. However, differences in sensitivity and specificity between the studies highlight the potential variations across populations of older females. Furthermore, discrepancies in the results between the two chair stand tests may be attributed to several factors. Although both tests exhibit a strong association, they likely assess different aspects of functional performance. The 5-CS test emphasizes dynamic balance and lower-limb strength, while the 30 s-CS test places greater demand on cardiovascular endurance due to its focus on sustaining repetitions over a timed 30-s period.

To our knowledge, previous studies have not extensively explored alternative functional tests such as the 30 s-AC and 30 s-CS for assessing slow gait speed. However, given the limited research on this type of analysis, we chose to dedicate a section to these findings. Our findings suggest that the 30 s-AC could be a valuable tool for identifying individuals with low gait speed (i.e., good sensitivity). In our sample, a cut-off value of 15.6 repetitions suggested low gait speed. While making direct comparisons with other studies is challenging, a previous investigation (34) found that achieving 11 repetitions in the 30 s-AC test was associated with a high risk of loss of functional independence among older adults of both sexes. It is important to note that our study focused on an older female population, with a slightly higher mean age (75 ± 6 years) compared to the referenced research (72 ± 8 years) (34). Despite this age difference, our participants showed a higher mean value (29.5%) on the arm curl test.

Overall, our findings showed varying sensitivities and specificities across different cut-off values, although the AUCs of the tests were similar. Among the functional tests, the 30 s-AC resulted in the highest AUC (0.80) and sensitivity (83%), but the lowest specificity (36%). This highlights the importance of prioritizing what is most crucial—whether it is more important to correctly identify individuals with slow gait speed (sensitivity) or to accurately classify those with normal gait speed (specificity). It is also crucial to assess the cost of misclassification and consider the potential consequences of incorrectly identifying individuals with good mobility as having low gait speed, or vice versa. This decision should be guided by the clinical context and screening objectives. Based on our results, the 30 s-CS, followed by the 5-CS, offered a good balance, with similar sensitivity but much higher specificity (ranging from 7% to 20%) compared to HGS and 30 s-AC.

We acknowledge that this study has some limitations in this study. First, as the data were collected outside the laboratory, we were unable to gather other relevant information on potential confounding factors, including muscle mass, lifestyle factors (e.g., physical activity levels or nutritional status), and anthropometric measurements (e.g., waist or hip circumference) or body composition indices. Notably, the lack of body composition measurements, such as fat distribution, appendicular lean mass, and lower extremity muscle mass, is a limitation. These parameters could have provided more comprehensive insights into the functional outcomes evaluated in this study. Additionally, while participants were interviewed to ensure they felt well and were fit for testing, no formal assessments for infections or fatigue on the testing day were conducted. Similarly, the intake of medications, drugs, or caffeine, which could potentially affect physical performance, was based on self-reports and not systematically documented. Furthermore, metabolic syndrome or obesity, which could have influenced the results, was not formally assessed. Second, our sample only included independent females, limiting the generalizability of our findings to the broader population or individuals with severe health conditions (e.g., a large sample of obese adults or those over 80 years old). Additionally, the absence of a male sample precludes us from drawing conclusions based on sex differences. Another notable limitation is related to the cut-off value for the gait speed used in our study. We were unable to test other commonly used thresholds, such as 0.8 m/s, due to our sample's limited number of participants with gait speeds below this threshold. It is important to note that our sample included females aged 60 and older, with the majority being in their early 70 s. Consequently, we described our sample as consisting of “youngest-older adults,” for whom a cut-off of <1 m/s is recommended (8). Hence, while this threshold provides a useful general guideline, its accuracy and applicability could vary and warrant further investigation in future studies.

In conclusion, our study found that HGS showed only a moderate-to-weak association with various functional outcomes in older females, suggesting that HGS alone may not always reflect lower body function. More importantly, we identified the 30 s-CS and 5-CS as potentially offering a good balance between sensitivity and specificity for detecting slow gait speed, which could have clinical implications for screening older adults at risk for mobility limitations. Our study provides specific cut-off values and quantitative associations that can guide clinical assessments and interventions in this population. These findings emphasize the importance of selecting functional tests based on the balance between sensitivity and specificity and tailoring specific clinical test choices to align with specific clinical objectives.

## Data Availability

The raw data supporting the conclusions of this article will be made available by the authors, without undue reservation.
